# Seminal plasma modulates the immune-cytokine network in the porcine uterine tissue and pre-ovulatory follicles

**DOI:** 10.1371/journal.pone.0202654

**Published:** 2018-08-28

**Authors:** Dagmar Waberski, Jana Schäfer, Alexandra Bölling, Manon Scheld, Heiko Henning, Nina Hambruch, Hans-Joachim Schuberth, Christiane Pfarrer, Christine Wrenzycki, Ronald H. F. Hunter

**Affiliations:** 1 Unit for Reproductive Medicine of Clinics / Clinic for Pigs and Small Ruminants, University for Veterinary Medicine Hannover, Hannover, Germany; 2 Institute for Anatomy, University for Veterinary Medicine Hannover, Hannover, Germany; 3 Immunology Unit, University for Veterinary Medicine Hannover, Hannover, Germany; 4 Clinic for Cattle, University of Veterinary Medicine Hannover, Hannover, Germany; University of Missouri Columbia, UNITED STATES

## Abstract

Evidence is emerging that the interaction between male seminal fluid and female tissues promotes fertility, pregnancy, and health of offspring. This includes the acceleration of ovulation in a species known as a spontaneous ovulator, the domestic pig. Earlier studies revealed that seminal plasma acts by a local mechanism in the female pig. The aim of the present study was to examine local short-term and mid-term effects of seminal plasma (SP) on mRNA expression of immunoregulatory genes and transcripts associated with follicle- and oocyte maturation. In the porcine animal model, effects on mRNA expression in the female tract and preovulatory follicles were examined. SP suppressed mRNA expression of Prostaglandin-Endoperoxide Synthase 2 (PTGS2) ipsilateral to the infused uterine horn which was associated with a lower presence of immune cells in the uterine epithelium and lower PTGS2 immunoreaction. Depending on the sampling time (2 h vs. 17 h) and hormonal status, SP altered significant correlative relations of mRNA expression between PTGS2 and the transcripts Tumor Necrosis Factor Alpha, Tumor Necrosis Factor Alpha-Induced Protein 6 and Pentraxin 3 in uterus, granulosa and cumulus cells. A modulatory effect of SP on the oocyte gene network comprising eight oocyte transcripts was observed: uterine exposure to SP induced positive correlations (r >0.08, p<0.05) of maturation promoting factors among each other and with cumulus cells on the side of the treated horn. In conclusion, SP orchestrates the gene network regulating the bidirectional communication between oocytes and surrounding somatic cells. The modulation of the immune-cytokine network of the female reproductive system could contribute to the previously reported SP-induced acceleration of ovulation in the porcine species.

## Introduction

Although it seems a little surprising that in vivo insemination as commonly used in human and animal assisted reproduction can give satisfying results with only residual seminal plasma (SP), there is now no doubt that the interaction of SP components with the female reproductive tract enhances the likelihood of successful establishment of pregnancy. Exposure of the uterus to SP triggers endometrial cells and resident leukocytes to synthesize chemokines and other mediators (reviewed in [1,2]). The ‘post-mating inflammatory response’ was classically described for the domestic pig by Lovell & Getty [3] and is now a widely-recognized feature of the post-coital uterus. Pro-inflammatory reaction involves an increased number of leukocytes infiltrating the uterine lumen, upregulation of MHC class II molecules and interleukin-2 (IL-2) receptor expression, and uterine expression of GM-CSF, IL-6, monocyte chemotactic protein- 1 (MCP-1) and PTGS2 [4,5,6].

While there has been substantial work on the uterine reaction to seminal fluid, until recently there has been only sparse information on ovarian responses. In a non-primate species also classified as a spontaneous ovulator, the domestic pig, seminal fluid contact accelerate ovulation [7], possibly by promoting leukocyte recruitment and with a subsequent advanced corpus luteum development [8]. Hence, the concept of ‘facultative-induced ovulators’ of all mammalian species, including human [9], was supported. It was then shown that the advancement of porcine ovulation predominantly resides in a local effect, as only the ipislateral ovary was affected when SP was deposited into one uterine horn only [10,11]. A further study suggested that SP induces an immediate and local cellular response in the uterus with a specific role of the utero-tubal junction [12]. Locally induced mediators, such as granulocyte-macrophage colony-stimulating factor (GM-CSF) and tumour necrosis factor-alpha (TNFα) [13], may reach the pre-ovulatory follicle, bind to receptors expressed on the surface of ovarian cells and modify follicular responses to gonadotropins (LH). Most notably, proinflammatory cytokine TNFα promotes ovulations in the rat ovary [14]. In pigs, a physiological routing of coitally-induced molecules would be possible via counter-current transfer from the uterine vein to the utero-ovarian artery and thus to the pre-ovulatory follicle [15]. It is now being increasingly recognized that ovulation involves coordinated and sequential inflammatory and innate immune responses. Function of the pre-ovulatory ovary is regulated by a plethora of locally induced cytokines mediating the paracrine dialogue between the oocytes and their surrounding somatic cells [16,17]. It is reasonable to propose that SP-induced cytokines modify the endocrine-immune-cytokine network in pre-ovulatory follicles leading to accelerated ovulation.

Beyond this background, we expand previous studies on the local effect using a porcine animal model allowing the unicornually application of SP whilst the contralateral horn served as control. The unique benefit of this model is having an animal as essentially its own control even though a systemic effect additionally to the previously described predominant local SP-effect [10,11] cannot completely ruled out. The above described evidence for possible signal routes in the female reproductive tract led us to hypothesize that SP modifies the local mRNA expression of immunoregulatory genes in the female reproductive tract which would then influence the gene network in the pre-ovulatory follicles of spontaneously cycling pigs. To test this hypothesis, gilts in oestrus were exposed to SP and mRNA expression of transcripts from selected cytokines and other mediators associated with follicle and oocyte maturation were determined in the uterus and different ovarian target cells. Since a SP response was expected to be time-dependent, two sampling times in oestrus were considered. Moreover, special attention was given to the potential regulation of gene expression at the utero-tubal junction.

## Material and methods

Unless otherwise stated all chemicals were obtained from Sigma (Steinheim, Germany).

### Animals

Sixteen hybrid gilts, aged 7–13 months, weighing between 140 and 180 kg were used in this study. All animals had experienced at least one spontaneous cycle prior to the experiment. They were housed in groups of two to three on straw in covered barns at the Unit of Reproductive Medicine, University of Veterinary Medicine Hannover. The gilts were fed a standard commercial diet of pellets and given free access to drinking water. The study was approved by the independent ethics committee of the Lower Saxony Federal State Office for Consumer Protection and Food Safety, Oldenburg, Germany (research permit number 33.12-42502-04-07/1261).

### Oestrus detection and sonography of ovaries

Studies were performed during spontaneous oestrus. Oestrus detection started at day 16 p.ov. by visual examination of the vulva and backpressure test in the presence of four different boars at intervals of four to six hours. The beginning of oestrus was set at the middle of the time interval between the last rejection and the first tolerance. From the 16^th^ day onwards, the ovaries were examined by transcutaneous sonography (5 MHz probe; Sonoline SI-400, Siemens) once daily, from the 18^th^ day on four times daily (05:00 h, 11:00 h, 17:00 h, 23:00 h) to determine the follicle size.

### SP preparation

Semen samples of ten mature boars with known fertility were collected over two months. The single ejaculates were centrifuged three times at 3.370 x g for ten minutes and the cell free plasma was stored frozen at -27 °C. The plasma pool was prepared by thawing the frozen samples, mixing and aliquoting in 100 ml bottles. Bottles were stored at -27 °C until use.

### Surgical approach and treatment

Uterine infusion of SP and phosphate buffered saline (PBS) were performed between 3 and 15 h after the calculated onset of oestrus under general anaesthesia. An intramuscular injection of atropine (Atropin sulfuricum^®^ sol. 1 vol%, Eifelfango, Bad Neuenahr; 0.02 mg/kg KGW i.m.) and a combination of ketamine–/ azaperone (Stresnil^®^, Janssen-Cilag GmbH; 2.0 mg/kg KGW i.m., Ursotamin^®^ Serumwerk Bernburg AG; 20 mg / kg KGW i.m.) were administered for premedication. Approximately 30 min later, anaesthesia was progressively deeper obtained by intravenous injection of ketamine (Ursotamin^®^) via an indwelling ear vein catheter. After endotracheal intubation, anaesthesia was maintained using a mixture of isoflurane, nitrous oxide, and oxygen. Throughout surgery, animals received an intravenous infusion of electrolytes (Albrecht GmbH, Aulendorf) and fundamental physiological variables were recorded using an anaesthesia monitor (Eagle 1000, Marquette Hellige).

Following aseptic procedures, the reproductive tract was exposed via a mid-ventral incision and, with the minimum handling of tissues, the number of mature pre-ovulatory Graafian follicles was recorded for each ovary. Double ligatures of an absorbable filament (Surgicryl^®^ PGA EP 6, SMI AG, Hünningen, Belgium) were positioned around each uterine horn approximately 10 cm distal to the uterine body. 100 ml of the 37 °C warm SP were slowly injected into one uterus horn and 100 ml 37 °C warm PBS into the contralateral horn using disposable sterile syringes, polyethylene extension and needle. An ovario-hysterectomy and a partial hysterectomy of 40 cm of the cranial uterine horns including the uterotubal junction was performed after 2 h (Experiment 1, n = 9 gilts) during the same surgical approach or after 17 h (Experiment 2, n = 7 gilts) at a second surgery. The mid-ventral incision was closed with three separate layers. Animal were returned to straw-bedded recovery boxes and received analgesic (Metacam^®^ Boehringer Ingelheim; 0.4 mg/kg KGW i.m.; Metamizol (Vetalgin^®^ Intervet, Unterschleißheim; 40 mg/kg KGW i.m.) and antibiotic (Amoxicillin, initially Hostamox^®^, 15 mg/kg KGW i.m., then Aciphen^®^ bela-pharm GmbH und Co. KG, Vechta; 2 x daily 20 mg/kg KGW orally) treatment.

### Blood sampling and hormone measurement

Peripheral blood samples were collected into tubes containing EDTA (Monovette^®^, Sarstedt, Nürnberg) immediately before treatment and immediately before ovariohysterectomy. Immediately after collection, blood samples were centrifuged at 3000 x g for 10 minutes and the plasma was stored at -27 °C until hormone analysis. Progesterone was measured by means of the competitive chemiluminescence-enzyme-immunoassay IMMULITE^®^ system (Siemens Diagnostics, Erlangen) according to the manufacturer’s instructions. The intra-assay coefficient of variance was 6.3%. Oestradiol was measured with an enzyme-immunoassay as described earlier [18,19]. The intra-assay coefficient of variance was 9.2%.

### Tissue and cell sampling

#### Uterine horns and uterotubal junction (UTJ)

Immediately after hysterectomy, the UTJ and pieces of each uterine horn which have been exposed either to SP or PBS were dissected, freed from surrounding mesometrium and washed with 37°C warm PBS. Tissue pieces were then divided lengthwise. One half was plunged into liquid nitrogen and stored frozen. The interval between tissue collection and fixation was at maximum 7 min. The other half was fixed by immersion in 4% buffered formalin and subsequently embedded in paraffin according to routine protocols.

#### Collection of epithelial cells via laser microdissection (LMD)

Slices of 8 μm thickness were cut from frozen tissue (cross sections) at -25 °C and subsequently stained with a modified haematoxylin–eosin stain as described by Fink & Bohle [20] with some modifications. Briefly, cryosections were mounted on polyethylene naphthalate membrane-covered glass slides (Membrane Slide 1.0 PEN, Zeiss, Göttingen). After staining with Mayer’s haematoxylin for 30 sec, the sections were rinsed for 20 sec in DECP water, then dipped in Eosin and subsequently immersed in 70%, 95%, and 100% ethanol. Laser microdissection was performed to isolate epithelial cells from tissue samples of uterine horns and uterine parts of the utero-tubal junction using the Leica LMD 7000 cryostat system equipped with the software Leica LMD n.v.6.6.3. Epithelial cells surrounding the uterine lumen were dissected at 10x magnification and immediately collected by gravitation in sterile DNase and RNase-free Eppendorf vials using the ‘cut and drop’ technique [21]. Dissected samples were stored at -80 °C up to seven days.

#### Oocytes, granulosa cells and cumulus cells

Immediately after ovario-ectomy, ovaries were placed in 37 °C warm PBS supplemented with 1% BSA and were transferred to the lab. Within 5 min after collection, granulosa cells, cumulus cells, and oocytes were separately collected from Graafian follicles with diameters > 5 mm: follicles were punctured individually with an insulin syringe and a small needle (22 G). The aspirated fluid was transferred into Tissue Culture Medium 199 (TCM 199) and granulosa cells and COC were isolated under a stereo microscope using two 22 G needles. The oocytes with residual cumulus cells were denudated with a stripper-pipette (Drummond Microdispenser, Broomall, USA) in several steps using three pipette tips (Mid Atlantic Diagnostics) of different diameter (290 μm, 135 μm and 125 μm). To ensure a good oocyte quality, denudation had to be finished within 30 minutes. Denudated oocytes were washed three times in 37 °C PBS with 1% PVA, transferred with a maximum of 1.5 μl PBS + PVA into a RNase and DNase free Eppendorf cup (Safe Seal reaction tube 0.5 ml, Sarstedt AG & Co., Nürnberg) and stored at -80 °C until analyses. In parallel with oocyte preparation, isolated granulosa and isolated cumulus cells of single follicles/oocytes were transferred into 50 μl TCM Air (1.51 mg TCM199, 5 mg gentamicin, 2.2 mg sodium pyruvate, 35 mg NaHCO_3_, 100 ml sterile water [Ampuwa*_*R, Fresenius, Bad Homburg, Germany], 100 mg BSA). 350 μl PBS was added and samples were centrifuged at 1000 x g for 10 min. Afterwards, the supernatant was discarded and the cell pellet was frozen at -80 °C.

### Histology and immunohistochemistry

Sections of 3–4 μm were produced from the paraffin embedded tissue and subjected to routine haematoxylin-eosin staining for general evaluation. Additionally, immunohistochemistry was performed with antibodies against neutrophil granulocytes and macrophages (MAC387, monoclonal mouse IgG, BioLogo, Kronshagen, Germany) and PTGS2 (monoclonal rabbit IgG, Thermo Fisher Scientific, Braunschweig, Germany). Briefly, sections were deparaffinized in xylol (2 x 10 min) and then rehydrated in a series of alcohols (isopropanol 2 min [only PTGS2], absolute alcohol (2 min). This was followed by incubation in a H_2_O_2_ solution (3%, 30 min), 70% alcohol (2 min) and a rinsing step in PBS (3 x 5 min). Then a pretreatment with TEC-buffer (pH 7.8, 30 min, 96–98 °C) was performed for PTGS2. Antibody MAC387 required treatment with Proteinase K solution (10 min, room temperature [RT], moist chamber). For all antibodies this was followed by rinsing in PBS (5 min) and incubation with 20% normal goat serum for 20 min. The primary antibody was diluted with PBS (+1% BSA) to a concentration determined in advance (MAC387 1:400; PTGS2 1:100) and incubated in a moist chamber at 4°C overnight. After rinsing the sections with PBS (3 x 5 min) they were incubated with secondary antibodies (goat-anti-mouse or goat-anti-rabbit 1:200 in PBS for 45 min at RT in a moist chamber). Another rinsing step in PBS followed (3 x 5min). Then the sections were subjected to the ABC system (ABC Vectastain Elite Kit) for 30 min at RT in a moist chamber and rinsed again in PBS (3x5min) before they were treated with the chromogen (DAB reagent 5 min, RT, moist chamber). After stopping the colour development by a rinsing step in PBS (1 x 5min) and running tap water (10 min), the sections were counterstained with hemalum for 5 sec. Finally, the sections were rinsed in running tap water for 15 min and dehydrated in graded alcohols (70% alcohol, 80% alcohol, absolute alcohol, isopropanol, each 2 min). They were mounted with Eukitt^®^ after immersion in Xylol (2 x 5min). Negative controls were performed by replacing primary antibodies with either PBS or rabbit or mouse IgG which were diluted like the primary antibodies.

From each animal one section from each localization was analysed in a blinded fashion.

Evaluation of the immunoreaction of the MAC387 antibody was conducted by light microscopy (Leica LMD 7000) at a magnification of x400 in two ways. For both, ten fields of view per section were analysed. For the locations subepithelial stroma, stratum compactum and reticulare, endometrial and perimetrial blood vessels, myometrium and perimetrium the amount of cells was assessed semiquantitatively (no cells, single cells, groups of cells, continuous presence of cells and massive infiltration) because counting was impossible due to high cell numbers.

For the PTGS2 antibody, in ten fields of view the staining intensity was classified in four categories (negative (= 0), weak reaction (= 1), positive (= 2) and strongly positive (= 3)) for the locations uterine epithelium, stratum compactum and reticulare, glandular epithelium, endometrial blood vessels and myometrial and perimetrial immune cells.

### RT-qPCR

#### Uterine epithelium, granulosa and cumulus cells

The mRNA expression of six immunoregulatory transcripts, *PTGS2*, Interleukin 6 (*IL6*), Pentraxin 3 (*PTX3*), Peroxisome Proliferator-Activated Receptor-Gamma 1 (*PPARG1*), Tumor Necrosis Factor Alpha (*TNFA*), Tumor Necrosis Factor Alpha-Induced Protein 6 (*TNFAIP6*) and Ubiquitin B (*UBB*) was analysed in epithelium of the uterine horn, the utero-tubal junction, granulosa cells and cumulus cells. To isolate RNA from the laser microdissected epithelium and the granulosa and cumulus cells stored at -80 °C, lysis buffer was added to the samples and RNA was extracted with the RNeasy^®^ plus micro Kit (Qiagen) according to the manufactures instructions. To ensure RNA quality and quantity, every single RNA sample was analysed with the Experion^®^ RNA Standard Sense Chips and the Experion^®^ RNA Standard Sense Analysis Kit^®^ (Bio-Rad, München, Germany) in the Experion^®^ Automated Electrophoresis System^®^ (Bio-Rad, München, Germany). To obtain same amounts of cDNA from each sample, RNA was diluted to 15 ng of total RNA/μl (epithelium of uterus and UTJ), 17 ng of total RNA/μl (granulosa cells) or 4.5 ng of total RNA/μl (cumulus cells).

Immediately thereafter, cDNA was generated by adding random primers and dnTPs (Invitrogen, Karlsruhe, Germany), water was added to a total volume of 13 μl. After incubation at 65 °C for 5 min, 5x First-Strand Buffer, 0.1 DTT, RNaseOUT^™^ Recombinant RNase Inhibitor and the reverse transcriptase Superscript^®^ III (Invitrogen, Karlsruhe, Germany) were added. The RT reaction was carried out for 5 min at 25 °C, 60 min at 50 °C and 70 °C for 15 min according to manufacturer’s instructions.

Messenger RNA copies were quantified with a Step One Plus real time PCR system (Applied Biosystems, Darmstadt, Germany). One microliter of the cDNA preparation representing 15 ng (epithelial cells), 17 ng (granulosa cells) or 4.5 ng (cumulus cells) were distributed to individual wells. Gene specific primer and the SYBR Green PCR Master Mix (Applied Biosystems, Darmstadt, Germany) were added. Primer sequences, product sizes (bp) and accession numbers are available (S1 Table). Water was added to a total volume of 25 μl. The PCR program started with denaturation at 95 °C for 10 min, followed by 40 cycles at 95 °C for 15 sec and 60 °C for 60 sec. Copy numbers of the transcripts were calculated from a dilution series (10^6^−100 copies) of the respective cDNA sub clones. Subsequently a melting curve was performed starting at 60 °C increasing the temperature every 15 sec for 0.3 °C to verify the amplified fragments.

#### Oocytes

Prior to RNA isolation, 1 pg of rabbit globin RNA was added as external control. Eight developmentally important transcripts, *c-Mos*, *CCNB1*, Mitogen-Activated Protein Kinase 1 (*MAPK1*), Cyclin-dependent Kinase 1 (*CDK1*), Proliferating Cell Nuclear Antigen (*PCNA*), Growth/Differentiation Factor 9 (*GDF9*), Bone Morphogenetic Protein 15 (*BMP15*) and Zygote Arrest 1 (*ZAR1*), were analysed in denuded and cryopreserved oocytes. Poly (A)^+^RNA was extracted as described by Stinshoff et al. [22] with the Dynabeads^®^ mRNA DIRECT^™^ Micro Kit (Invitrogen, Karlsruhe, Germany) and directly used for reverse transcription (RT). RT was carried out in a total volume of 20 μl. The reaction mix contained 1x PCR Buffer (Invitrogen, Karlsruhe, Germany), 5 mM MgCl_2_ (Invitrogen, Karlsruhe, Germany), 1 mM dNTPs (Eurogentec, Köln, Germany), 2.5 μM random hexamers (Applied Biosystems, Darmstadt, Germany), 20 U RNase inhibitor and 50 U murine leukaemia virus (MuLV) reverse transcriptase (Applied Biosystems, Darmstadt, Germany). The RT reaction was carried out at 25 °C for 10 min, 42 °C for 60 min followed by denaturation at 99 °C for 5 min and was then stored on ice.

The relative abundance of mRNA was measured in each individual oocyte in the IQ5 Multicolor Real-Time PCR Detection System (Bio-Rad, München, Germany). The PCR reaction mix contained 10 μl Mastermix (dNTPs, Taq-Polymerase, MgCl_2_, SYBR^®^ Green, stabilizer, fluoreszin), 0.2 μM primer forward and reverse each for the gene of interest and the external reference gene (Eurofins MWG, Ebersberg, Germany; S2 Table). It also contained cDNA varying from 0.5 to 2 μl oocyte equivalent of each oocyte. Water was added to a total volume of 20 μl. The PCR program started with denaturation at 95°C for 10 min, followed by 43 cycles at 95 °C for 15 sec, 60 °C for 30 sec and 72 °C for 30 sec. Subsequently a melting curve was performed starting at 55 °C and increasing the temperature every 10 sec for 0.5 °C to verify the amplified fragments. The level of transcript expression was calculated using the external control (rabbit globin) for relative quantification. During the log-linear phase of amplification, the number of threshold cycles (CT) required for each product to exceed background fluorescence was recorded. In order to assess the relative abundance of gene transcripts a method proposed by Pfaffl [23] was used. Briefly, the comparison among groups was made possible by using the ΔΔCT approach to assess differences in fold change. For this the efficiency range between target and the exogenous control had to be between 95 and 100%. The chosen approach is in accordance with Schmittgen & Livak [24].

### Statistical analysis

Data were analysed using the Statistical Analysis Software SAS 9.3 (SAS Institute Inc., Cary, North Carolina, USA). A gene was only considered for statistical analysis if its mRNA expression levels were above detection level (> 100 copies) in samples from both sides of the genital tract in more than 50% of the animals (c.f. S1 Table). Data were tested for normal distribution using the Shapiro-Wilk-Test (PROC UNIVARIATE). In general, data were not normally distributed. Logarithmic transformation resulted in normal distribution only for mRNA copy numbers of selected genes in samples from granulosa cells of animals with low or high E/P4 ratio. Consequently, a Student’s t-test (PROC UNIVARIATE) was used for comparing mRNA levels between both groups. For all other comparisons the Wilcoxon’s signed rank test was used (PROC UNIVARIATE).

The mRNA copy numbers in samples from the uterus, the utero-tubal junction, granulosa cells, and cumulus cells showed high variations between animals for any given gene. In order to reduce the bias of the high inter-animal variations and achieve a more standardized comparison, coefficients of relative differences in mRNA expression were calculated, when testing for differences in mRNA levels between e.g. the SP-treated and PBS-treated side or between locations within the SP-treated or PBS-treated side of the genital tract.

An exemplary calculation is given in Eq 1.

$$coefficient\;=\;\frac{mRNA\;copy\;number\;(SP-treated\;side)\;}{mRNA\;copy\;number\;(PBS-treated\;side)}-1$$ (1)

The coefficient from the example is used for comparing the mRNA expression level at a given location (e.g. uterus) between the SP-treated side and the PBS-treated side of the genital tract. The coefficients were entered into the calculations for the statistical tests (Student’s t-test and Wilcoxon’s signed rank test) instead of the commonly used differences (deltas) in mRNA copy numbers. Both, Student’s t-test and Wilcoxon’s signed rank test are testing the hypothesis that no significant deviation from zero exists for a given set of numbers. If the hypothesis was accepted, no significant difference in mRNA expression levels was assumed. If the hypothesis was rejected, a significant difference in mRNA expression levels was assumed. Data are reported as original mRNA copy numbers in all tables and figures.

Correlations between mRNA copy numbers of selected transcripts within a location or between locations were calculated with Spearman’s rank correlation coefficient (PROC CORR). Data of neutrophils in the uterine epithelium were analysed using Wilcoxon signed-rank test (not normally distributed data).

Differences were considered statistically significant if P < 0.05.

## Results

### Reproductive tracts and hormonal status

All animals exhibited signs of oestrus including hyperaemic, oedematous vulvae and had normally developed reproductive tracts. Ovarian follicles ranged between 7 and 11 mm in diameter and residual corpora lutea were present from the previous cycle. The median of the relation between oestradiol and progesterone at the time of treatment was 15.9 (range: 4.1 to 81.5; Groups 1 and 2, n = 16 gilts). In Group 2, the hormonal status was additionally examined at the time of tissue sampling, i.e. 17 h after treatment: the median of oestradiol and progesterone was 11.5 (range: 9.2 to 20.8).

### Effect of SP on immune cells and PTGS2 immunoreaction in the uterine epithelium

**Fig 1:**
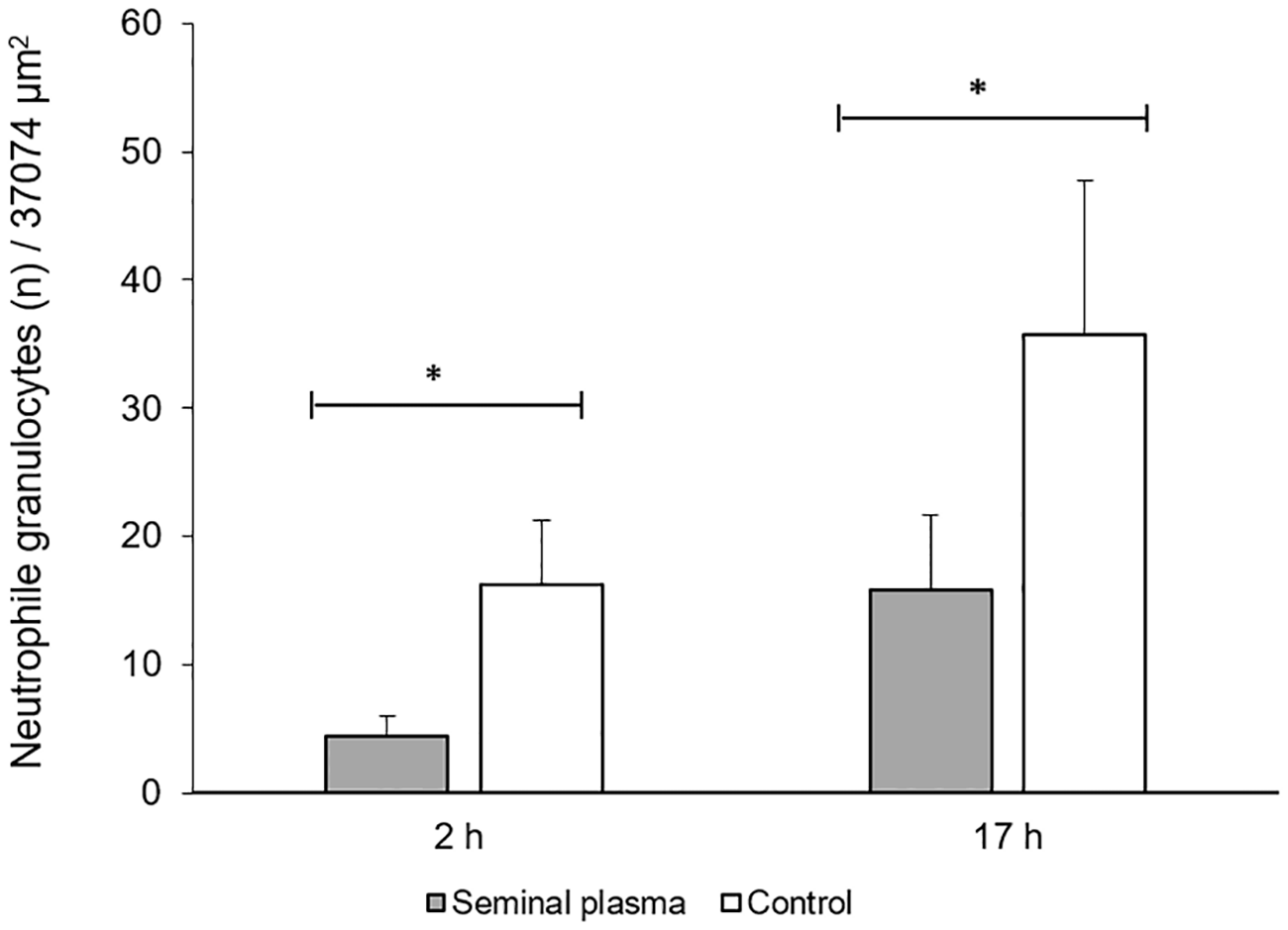
Neutrophile granulocytes (n) in epithelium of uterine horns at 2 h (Group 1, n = 9 gilts) and at 17 h (Group 2, n = 7 gilts) of seminal plasma and contralateral infusion of PBS (control). Asterisks indicate significant differences (P< 0.05).

**Fig 2:**
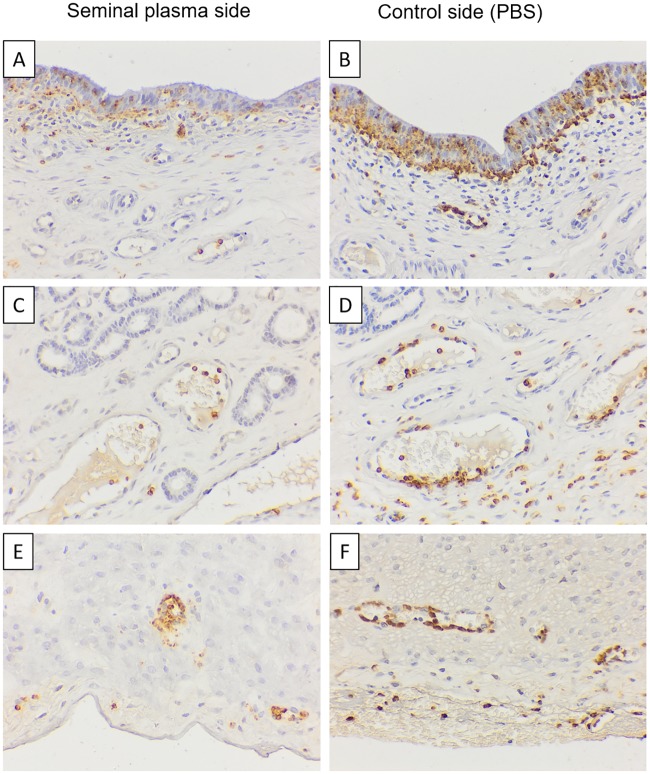
Immunhistochemical staining with antibody MAC387. Representative images of an animal at 17 h after single uterine infusions. There are less neutrophils (brown stained) in the layers epithelium (A, B), stroma (C, D) and serosa (E, F) of the uterine horn infused with seminal plasma plasma (A, C, E) than the one infused with PBS (B, D, F). Bar = 40 μm. Ep: Epithelium; Gl: Glandular; Se: Serosa, Asterisk: blood vessel.

The number of neutrophils in the uterine epithelium was statistically significantly lower (p< 0.05) in the SP treated side when compared to PBS infused sides (Fig 1). Semiquantitative analysis of the other uterine tissues also revealed less neutrophils in the stroma and serosa of SP treated sides (Fig 2).

**Fig 3:**
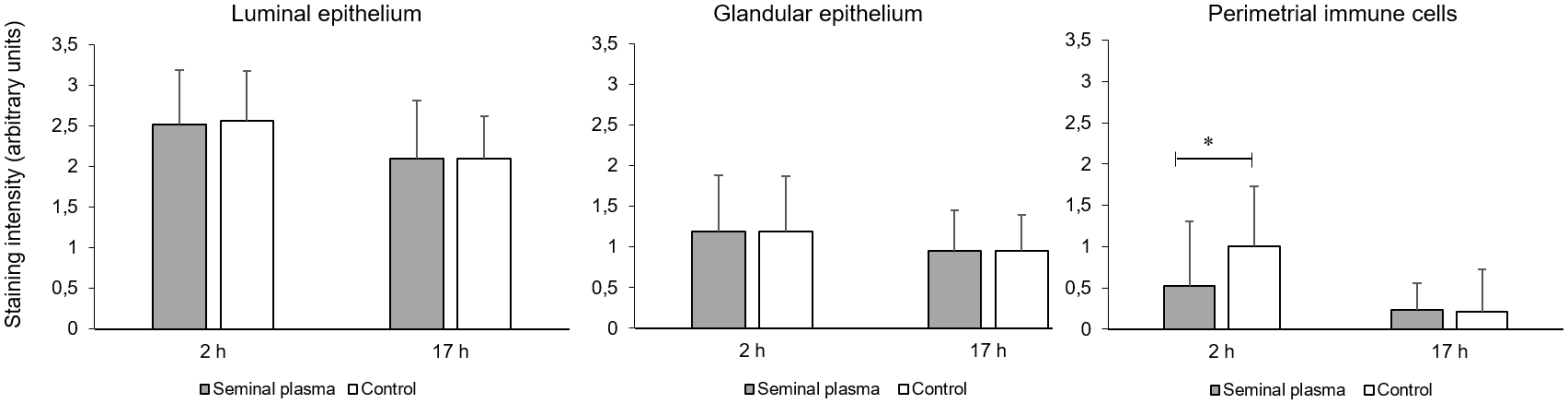
Immunhistochemical staining intensity of PTGS2-positive immune cells in different uterine cell types at 2 h (Group 1, n = 9 gilts) and at 17 h (Group 2, n = 7 gilts) after single uterine horn infusions of seminal plasma and contralateral infusion of PBS. Stain scores: 0 = negative, 1: weak, 2 = positive, 3 = strongly positive). Asterisk indicates a significant difference (P< 0.05).

PTGS2 immunoreaction was higher in luminal epithelium of uterine horns compared to glandular epithelium (Fig 3). Additionally, in Group 1 animals (2 h after infusion), perimetrial immune cells showed a lower staining intensity in SP treated sides compared to the PBS controls.

### Messenger RNA expression at different locations of the reproductive tract

**Fig 4:**
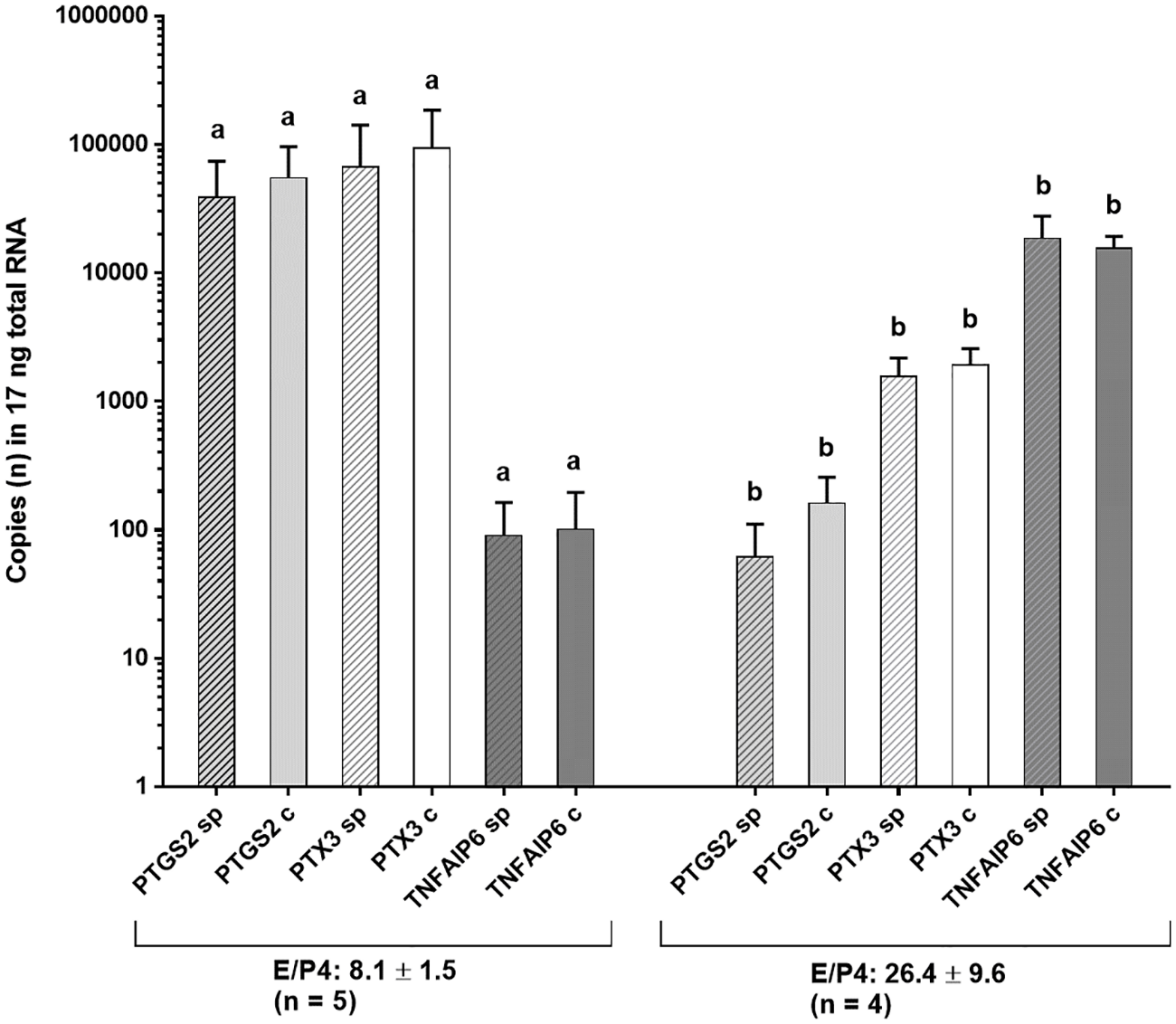
Messenger RNA-expression of the transcripts PTGS2, PTX3 and TNFAIP6 in 17 ng total RNA of granulosa cells at 2 h after single uterine horn infusion of seminal plasma and contralateral infusion of PBS (control) in subgroups of gilts with low E/P4 ratio and high E/P4 ratio (means and SEM). a, b: values between subgroups of sows are significantly different (P < 0.05).

The number of animals with mRNA expression (> 100 copies) of the cytokine genes *IL6*, *TNFA* and *TNFAIP*, of the receptor proteins *PPARG1* and *PTX 3*, and of the enzyme *PTGS2* and of *UBB* as reference at the different location of the female tract both on treated and control sides is shown in Table 1. Messenger RNA of the transcripts *PTGS2*, *TNFAIP6* and *UBB* was consistently expressed in all tissue samples in most animals. *IL6* mRNA expression was found in the majority of gilts in the uterine epithelium in both groups of gilts and in granulosa cells of most animals in Group 2. No gene expression of *IL 6* was detected in cumulus cells. Messenger RNA of *TNFA* was expressed in both epithelium of uterine horns and UTJ, but not in granulosa and cumulus cells. *PPARG1* mRNA was expressed in all animals in granulosa cells only. Expression of the transcript *PTX3* was consistently found in granulosa and cumulus cells, but only irregularly in uterine samples. Gene expression levels varied between animals. In Group 1 (sampling 2 h after treatment), expression levels in granulosa cells were related to the hormonal status (Fig 4). Animals displaying high mRNA expression levels of transcripts *PTGS* and *PTX3* in granulosa and cumulus cells (> 50,000 copies in 17 ng total RNA, n = 5 gilts) had significantly lower oestradiol/progesterone ratios compared to animals with low expression of these transcripts (< 5,000 copies in 17 ng total RNA, n = 4 gilts). Time or type of treatment did not influence the distribution of gene expression in the different tissue samples (summarized data in S3 Table).

**Table 1:** Absolute copy numbers in RNA of uterus epithelium, granulosa and cumulus cells at 2 h (A, Group 1, n = 9 gilts) and at 17 h (B, Group 2: n = 7 gilts) after single uterine horn infusion of seminal plasma (SP) and contralateral infusion of PBS (Control = C); Means ± SEM.

A) 2 h	PTGS2		IL6		PPARG1		PTX3		TNFA		TNFAIP6		UBB	
Localisation	SP	C	SP	C	SP	C	SP	C	SP	C	SP	C	SP	C
Uterine horn*	16256 ± 4787	16995 ± 2519	170 ± 24	184 ± 18	< 100 ±	< 100	< 100	< 100	1240 ± 360	893 ± 329	526 ± 108	455 ± 95	106771 ± 15108	80480 ± 5664
Utero-tubal Junction*	4128 ± 2609	10271 ± 3299	185 ± 60	211 ± 22	< 100	< 100	< 100	< 100	315 ± 80	452 ± 147	191 ± 29	296 ± 56	93193 ± 11831	93636 ± 7483
Granulosa cells**	45596 ± 15201	52563 ±21808	< 100	< 100	740 ± 261	720 ± 207	75914 ± 23078	100239 ± 33035	< 100	< 100	14417 ± 8205	9164 ± 6873	80118 ± 11211	72933 ± 6800
Cumulus cells***	4449 ± 1774	4662 ± 2062	< 100	< 100	143 ± 35	183 ± 46	10519 ± 5344	7617 ± 2297	< 100	< 100	7679 ± 6209	3513 ± 1882	20089 ± 3650	20545 ± 4669
**B) 17 h**														
Uterine horn*	5633^a^ ± 1375	11880^b^ ± 2842	< 100	< 100	< 100	< 100	< 100	< 100	1006 ± 324	1409 ± 221	472 ± 136	677 ± 186	49941 ± 13344	53543 ± 8916
Utero-tubal Junction*	2663 ± 699	6070 ± 2988	< 100	< 100	< 100	< 100	< 100	< 100	323 ± 90	519 ±172	323 ± 90	519 ± 172	57645 ± 15226	37777 ± 9352
Granulosa cells**	56414 ±12794	57262 ±13781	< 100	< 100	422 ± 84	408 ± 50	174506 ±14924	182682 ±32869	< 100	< 100	< 100	< 100	82572 ±15435	68837 ± 11347
Cumulus cells***	16036 ± 3937	14778 ± 3805	< 100	< 100	< 100	< 100	25511 ± 4949	33045 ± 6358	< 100	< 100	1762 ± 674	624 ± 143	18388 ± 2690	17288 ± 2342

^a, b^: Significantly different (p< 0.05)

* 15 ng total RNA;

** 17 ng total RNA;

*** 4.5 ng total RNA

### Effect of SP on mRNA expression at different locations of the female tract

**Fig 5:**
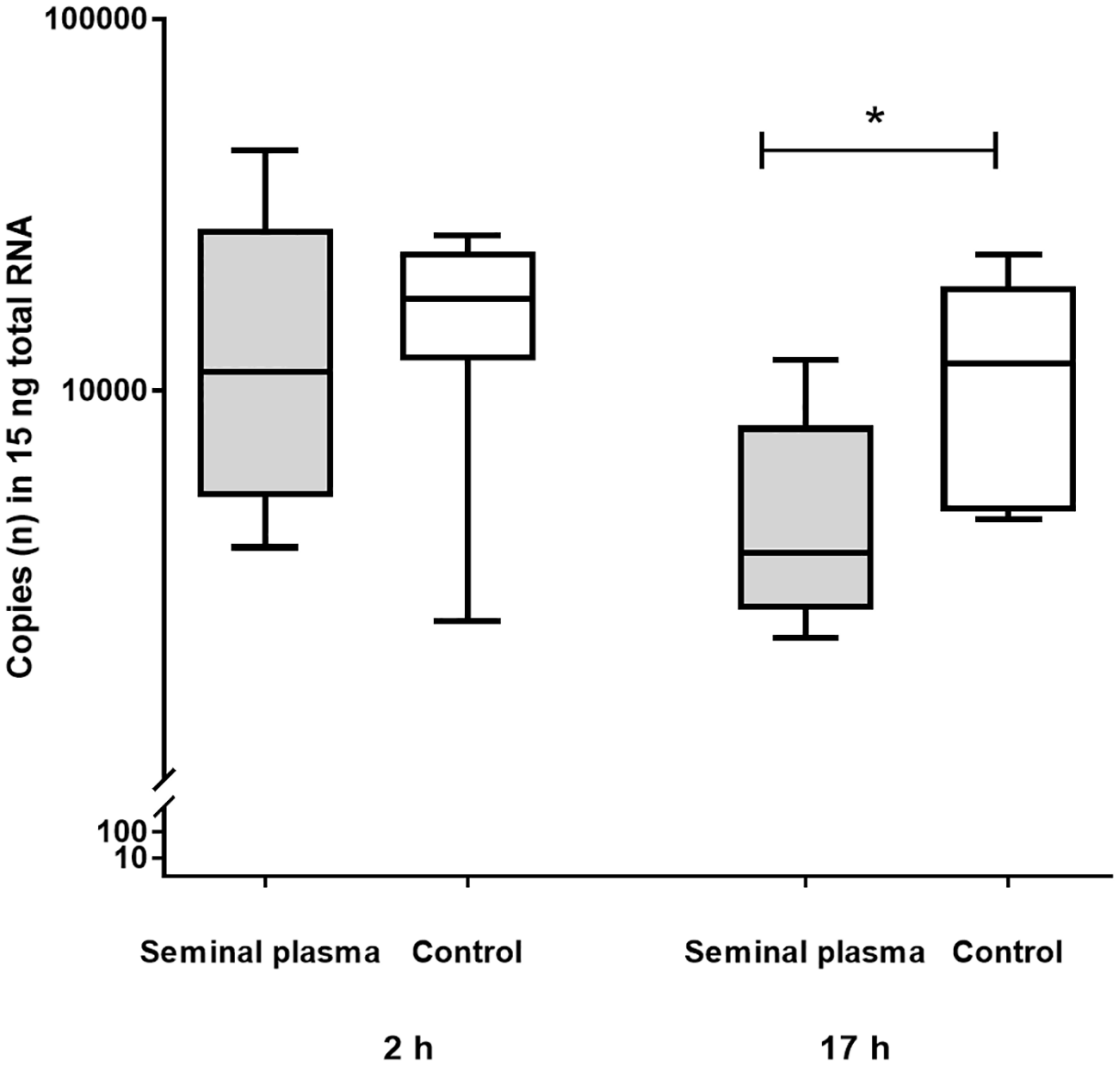
Messenger RNA expression of PTGS2 in epithelium of uterine horns at 2 h (Group 1, n = 9 gilts) and at 17 h (Group 2, n = 7 gilts) after single uterine horn infusion of seminal plasma and contralateral infusion of PBS (Control). Asterisk indicates a significant difference (P < 0.05).

Messenger RNA expression on treated and control sides at the different locations is shown for both groups of gilts in Table 1. Gilts in Group 1 (sampling at 2 h after treatment) did not show significant differences of gene expression levels between treated and control sides. Gilts in Group 2 (sampling at 17 h after treatment) revealed lower (P < 0.05) mRNA expression of *PTGS2* in the epithelial cell layer of uterine horns treated with SP compared to control horns (Fig 5). No further difference between gene expression levels on treated and control sides was found.

### Comparison of mRNA expression levels between uterine horns and UTJ

**Fig 6:**
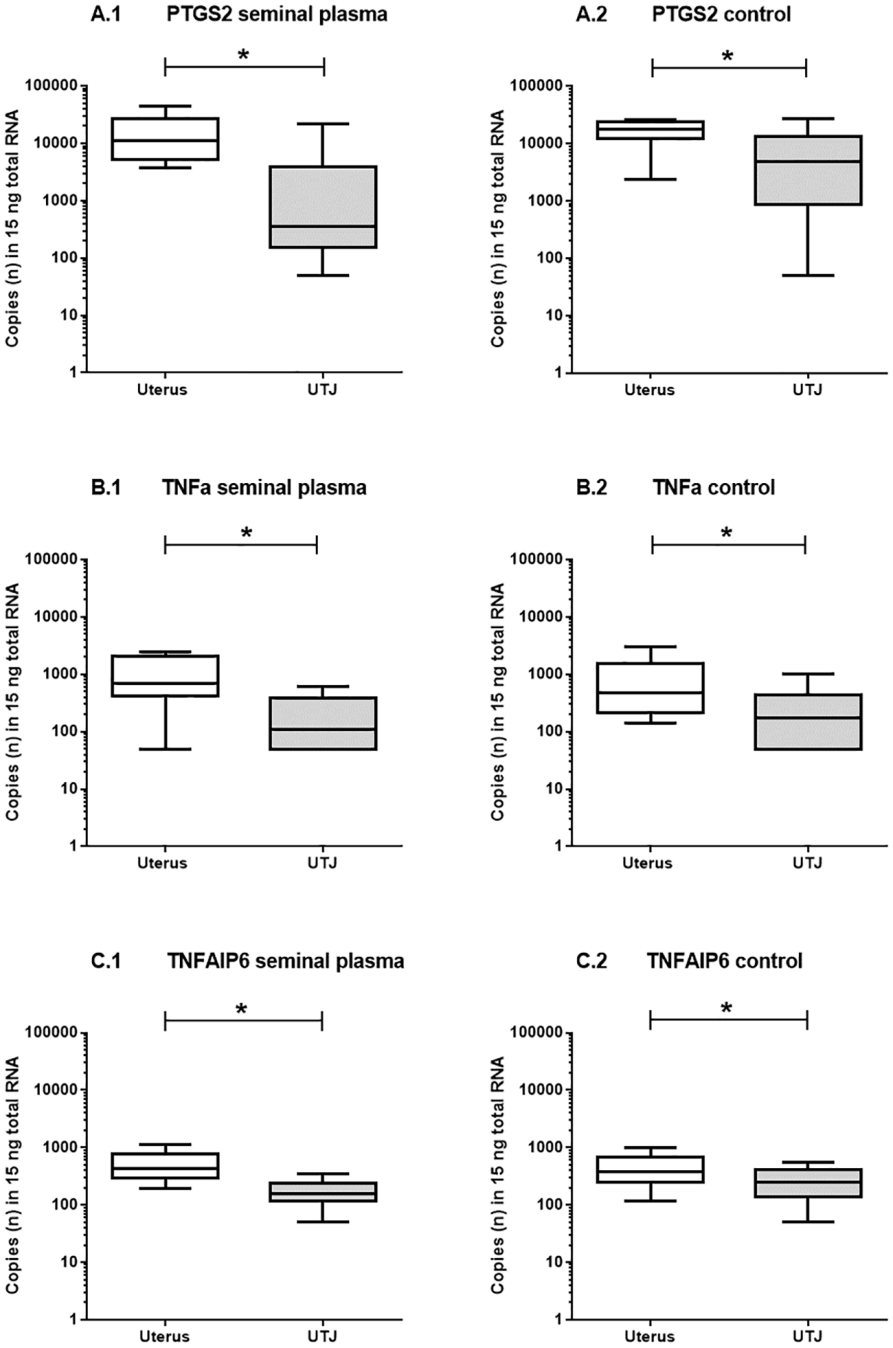
Messenger RNA expression of the transcripts PTGS2 (A), TNFA (B) and TNFAIP6 (C) in 15 ng total RNA of epithelia cells from uterine horns and utero-tubal junction (UTJ) after single uterine horn infusion of seminal plasma and contralateral infusion of PBS (control) at 2 h after infusion (n = 9 gilts). Asterisks indicate significant differences between the locations (P < 0.05).

In both groups of gilts, mRNA expression of *PTGS2*, *TNFA* and *TNFAIP6* was higher (P < 0.05) in the epithelium of uterine horns compared to the uterine part of the UTJ. Differences were observed on both treated and control sides. Data are presented for animals in Group 1 in Fig 6. Gene expression of *UBB* did not differ between the two locations.

### Correlation of mRNA expression levels between transcripts at different locations of the reproductive tract

At 2 h after treatment (Group 1), mRNA expression was highly correlated between uterine horns and adjacent UTJ for the transcripts *PTGS2* (r = 0.8, P < 0.01), *TNFA* (r = 0.9, P < 0.001) and *TNFAIP6* (r = 0.9, P < 0.001) on the sides treated with SP, but not on contralateral sides. At 17 h after treatment (Group 2), no significant correlations were found.

In animals of Group 1, gene expression of *TNFA* in the UTJ correlated significantly with expression of *PTGS2* (r = 0.7, P < 0.05) and *PTX3* (r = 0.7, P < 0.05) in granulosa cells ipsilateral to uterine horns treated with SP, but not on control sides (Table 2). Such correlations were not found in Group 2. No correlations were found between gene expression levels of transcripts in the UTJ and cumulus cells in any of the groups. In Group 1 (2 h after infusion), mRNA expression of *PTX3* in granulosa cells was highly positively correlated with mRNA expression levels of *PTGS2* in granulosa cells (r = 0.99, P < 0.05) and cumulus cells (r = 0.82, P < 0.05) on both sides of the female tract. Data for both groups of gilts are presented in S4 Table.

**Table 2:** Correlations (r, p<0.05) between mRNA expression levels of transcripts in the utero-tubal junction and granulosa cells on the side of the SP-treated uterine horns at 17 h after infusion (Group 2, n = 7 gilts).

	Granulosa cells		
	PTGS2	PPARG1	PTX3
Utero-tubal junction	r	r	r
PTGS2	n.s.	n.s.	n.s.
TNFA	0.70	n.s.	0.70
TNFAIP6	n.s.	n.s.	n.s.

n.s.: not significant (P>0.05)

No correlations (p>0.05) were found on the side of PBS-treated uterine horns in the same animals.

### Messenger RNA expression in oocytes

Messenger RNA expression of all eight transcripts associated with follicle growth and oocyte maturation was found in oocytes from both groups of animals. Relative amounts of transcript mRNA expression varied between gilts, but were correlated (r > 0.8, P < 0.05) between treated and control sides for all genes of interest, except for ZAR1. The relative mRNA expression levels did not differ between treated and control sides (S5 Table). At 2 h after treatment (Group 1) correlations between mRNA expression of *PTGS2* in cumulus cells and that of three oocyte transcripts *PCNA*, *GDF9* and *BMP15* as well as between *PTX3* mRNA expression in cumulus cells and six oocyte transcripts *PCNA*, *GDF9* and *BMP15*, *c-Mos* and *CCNB1*, were found on the sides of SP-treated uterine horns (Table 3A); no correlations were established on control sides. In this group, no significant correlations were found between mRNA transcript expression in granulosa cells and oocytes. At 17 h after treatment (Group 2), mRNA expression of *TNFAIP6* in granulosa cells was negatively associated with relative amounts of five oocyte transcripts (Table 3B). No significant correlations were found on control sides, and between mRNA transcript expression in cumulus cells and oocytes.

**Table 3:** Correlations (r, p<0.05) between mRNA expression levels of transcripts in somatic ovarian cells and relative amounts of oocyte transcripts on the side of the SP-treated uterine horns.

	2 h after SP infusion (Group 1, n = 9 gilts)			17 h after SP infusion (Group 2, n = 7 gilts)		
	Cumulus cells			Granulosa cells		
	PTGS2	PTX3	TNFAIP6	PTGS2	PTX3	TNFAIP6
Oocytes transcript	r	r	r	r	r	r
ZAR 1	n.s.	n.s.	n.s.	n.s.	n.s.	n.s.
c-Mos	n.s.	0.86	n.s.	n.s.	n.s.	n.s.
MAPK1	n.s.	n.s.	n.s.	n.s.	n.s.	n.s.
Cyclin B1	n.s.	0.79	n.s.	n.s.	n.s.	-0.74
CDK1	n.s.	0.79	n.s.	n.s.	n.s.	-0.88
PCNA	0.82	0.90	n.s.	n.s.	n.s.	-0.79
GDF9	0.86	0.86	n.s.	n.s.	n.s.	-0.79
BMP15	0.82	0.90	n.s.	n.s.	n.s.	-0.78

No correlations (p>0.05) were found on the side of PBS-treated uterine horns in the same animals.

n.s.: not significant (P>0.05)

### Correlation of mRNA expression levels in oocyte transcripts

**Fig 7:**
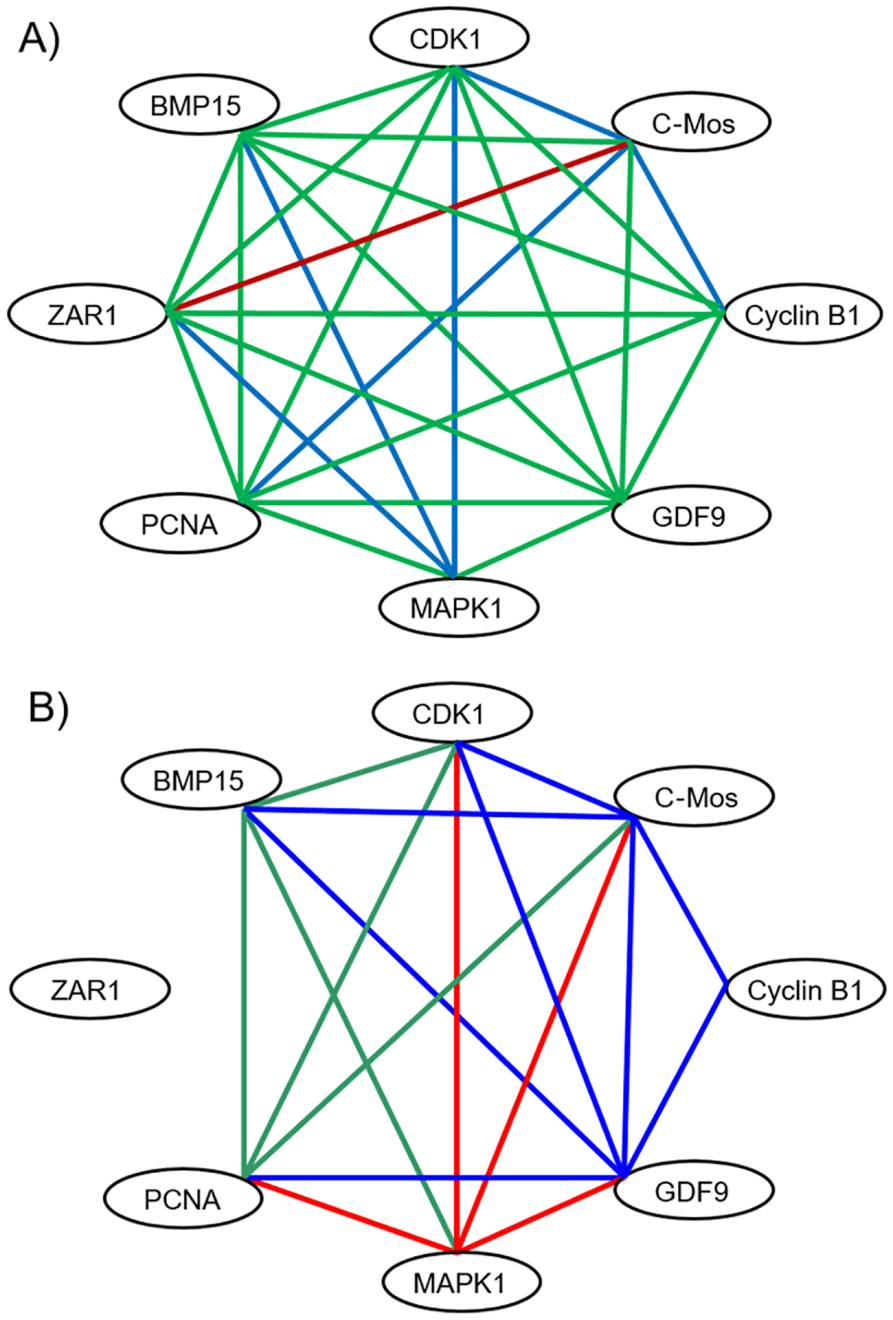
Oocyte gene network based on mRNA expression of transcripts associated with follicle growth and oocyte maturation at 2 h (Group 1; A, n = 9 gilts) and at 17 h (Group 2; B, n = 7 gilts) after single uterine horn infusion of seminal plasma and contralateral infusion of PBS (Control). Lines indicate significant positive correlations between transcripts with r ≥ 0.70, P < 0.05. Green lines: correlation in ovaries on the side of both SP- and PBS-treated uterine horns. Blue lines: correlation in ovaries on the side of SP-treated uterine horns only. Red lines: correlation in ovaries on the side of PBS-treated uterine horns only.

At 2 h after treatment (Group 1), relative mRNA expression levels of most transcripts were highly correlated (r ≥ 0.7, p < 0.05) to each other on ovaries from both control and treated sides. Only on the side of the treated uterine horn, relative expression levels of *MAPK1* were positively correlated with mRNA–expression of *CDK1*, *BMP 15* and *ZAR1*, and relative expression levels of *c-Mos* were positively correlated to those of *CCNB1*, *PCNA*, *CDK1* (Fig 7A). At 17 h after treatment (Group 2), only few correlations were found between relative expression levels on oocytes of from the treated and control side (Fig 7B). On the side with uterine infusion of SP, relative amounts of the transcript *GDF9* were positively correlated with five other transcripts, i.e. *PCNA*, *BMP15*, *CDK1*, *C-Mos*, and *CCNB1* and relative amounts of the transcript c-Mos were positively correlated with four other transcripts, i.e. *CDK1*, *BMP15*, *GDF9*, and *CCNB1*. On the control side, only MAPK1 expression levels were positively correlated with four other transcripts (*PCNA*, *CDK1*, *C-Mos*, and *GDF9*).

## Discussion

The identification of relationship among genes, proteins or molecules in biological pathways is critical for understanding complex biological activities and biological functions [25]. The present study reveals a novel biological pathway by demonstrating that SP modulates the immune-cytokine network in the uterine tissue and ovaries. Our data show that the interaction network of oocyte factors based on their correlation of mRNA expression levels is altered by uterine infusion of SP. This effect was most obvious at the more advanced oestrus (17 h after treatment) with *GDF9* and *c-Mos* as key factors showing most correlative relations with other transcripts. At 2 h after treatment, the interaction network between the transcripts of the eight studied oocyte factors seems to be denser regardless of the treatment. At this early time, the modulatory influence of SP induces positive correlations of maturation promoting factors including *MAPK1* among each other and with other oocyte factors. The presence of high positive correlation of mRNA expression of the two oocyte-secreted growth factors *GDF9* and *BMP*15 with mRNA molecule concentrations of both *PTGS2* and *PTX3* in cumulus cells only on the side of SP-treated uterine horns indicates that SP modulates the interaction of the different cell types in antral follicles. There is strong evidence that fine gene dosage mechanisms involving GDF9 and BMP15 play an important role in the regulation of ovulation across mammalian species including human [26,27]. In the present study, high expression of oocyte maturation-associated transcripts *BMP15*, *GDF9*, *CCNB1*, *CDK1*, *PCNA* and low expression levels of *TNAIP6* in granulosa cells indicate an advanced oestrous stage approaching ovulation. The observed unilateral negative correlation between *TNFAIP6* in granulosa cells and maturation associated oocyte transcripts at 17 h after treatment indicates that SP advances the synchronization of mRNA expression between the different cell types in the preovulatory follicle. Thus, we suggest that SP facilitates ovulation by influencing the oocyte-CC regulatory loop, at least in porcine species where large amounts of SP enter the female tract and spontaneous ovulation would only occur in the last third of the oestrus period. The effect of male genital fluids will inevitably enhance fertilization chances by preventing loss of sperm quality associated with ageing in the female tract. Whether exposure to SP also would affect oocyte quality is as yet unknown. The observation that SP induces a correlative relationship between *PTX3* in cumulus cells and mRNA expression of regulators of oocyte maturation *c-Mos*, *CCNB1 CDK1* in addition to *BMP15* and *GDF9* supports this hypothesis because these maternal transcripts as well as relative abundance of *PTX3* mRNA in cumulus cells are considered as markers for development competence of female germ cells in human, pig and mouse [28,29].

At 2 h after single uterine horn infusion of SP, expression of *PTX3* mRNA in granulosa cells was ipsilateral highly positively correlated with mRNA expression of *PTGS2* in cumulus cells. Cumulus cells similar to granulosa cells express numerous immune cell-related genes and are very sensitive to changes in the immediate environment [16]. Since cumulus cells were collected from intraovarian COC’s, a direct contact to SP or to SP-primed tissue and extraovarian immune cells could be excluded. It rather seems that SP orchestrates the innate immune surveillance system in ovarian cells through sequential modulation of immune-response-related genes in the female reproductive tract including the bidirectional communication between oocytes and surrounding somatic cells. Granulosa and cumulus cells express innate immune characteristics and respond to systemic or local stimuli with the release of potent cytokines and chemokines involved in COC expansion, synthesis of a hyaluronan-rich matrix and ovulation [30]. Systemic hormonal stimuli of gonadotropic hormones interact with local stimuli thereby orchestrating periovulatory events in the Graafian follicles. In the present study, the time of the endogenous LH-surge was unknown, but mRNA expression levels of matrix-associated molecule genes *PTGS2*, *PTX3* and *TNFAIP6* in granulosa cells were associated with different oestradiol/progesterone ratios thus explaining the inter-individual variations of mRNA expression. Animals with low oestradiol/progesterone ratios indicating an oestrus stage closer to ovulation exhibited higher expression of *PTGS2* mRNA. This result corresponds with kinetic changes of pre-ovulatory gene expression in granulosa and cumulus cells of sows primed with hCG [31].

A steep increase in prostaglandins, principally PGE2, in the pre-ovulatory follicle shortly before follicle rupture, is a key event in ovulation [32]. A local influence of SP at 2 or 17 h post infusion was not detected in mRNA expression of granulosa and cumulus cells, presumably because at early oestrus expression of *PTGS2* mRNA is predominantly controlled by LH and steroid hormones [31]. In fact, uterine application of boar SP at the onset of oestrus accelerated ovulation in spontaneously cycling animals with a longer oestrus duration [11] but failed to advance ovulation in hCG-treated sows [33]. Thus, the orchestrating effect of SP on oocyte gene interaction is under superordinated control of gonadotropic hormones and therefore the effect of SP depends on the time of treatment and sampling, and the time-span between the two. A time-dependency in the response to boar SP was indicated in several previous in vivo studies measuring the mRNA expression patterns of immune-related endometrial transcripts [5,6]. In the present study, a lower the mRNA expression of *PTGS2* in the uterine epithelium was observed at 17 h after SP infusion compared to control which could be explained by the concomitant lower number of neutrophils. Resident immune cell and their interaction with epithelial cells are most likely to mediate local immune effects. In line with the present results, it was reported that porcine SP inhibits neutrophil immigration, accelerates PMN clearance from the uterus and suppresses chemotaxis of PMN (induced cycles: [34,35,36]; spontaneous cycles: [37]. Other immune cells may also contribute to the SP response as indicated by a lower PTGS2 immunoreaction in perimetrial immune cells of treated uterine horns.

Earlier experiments with a similar porcine in vivo model demonstrated that the contact of SP with the uterotubal junction is essential for the advancement of ovulation [38] and may be related to an elevated number of MHC class II positive cells [12]. Messenger RNA expression of the selected transcripts studied here was higher in the uterine horns compared to the epithelium of the utero-tubal junction, supporting a specific role of the UTJ with an autonomous regulation of transcript expression by the epithelial cells or by a different population of resident cells. Positive correlations found in mRNA expression on SP-treated sides support this view.

Similarly to the biological pathway reported here, in cows with inflamed uteri post partum, a localized effect on ovaries ipsilateral to the previously gravid uterine horn has been demonstrated: fewer first and second dominant follicles were selected and fewer first dominant follicles ovulated in the ipsilateral compared with the contralateral ovary, respectively. Accordingly, cows with uterine infection exhibit delayed ovulation in vivo [39,40]. These reports together with observations presented here offer a new hypothesis on the effect of SP on the time of ovulation in pigs: Given that insemination causes a local inflammatory reaction stimulated by a volume effect and a plethora of cells acting as antigen, immunosuppressive effects of SP could counteract adverse effects on folliculogenesis which otherwise would delay ovulation.

Under conditions of natural mating or insemination, the interaction between the different cell types will be further modified by the presence of sperm. Spermatozoa modulate mRNA concentrations of cytokines acting as pro- and anti-inflammatory mediators in porcine species at 3 h after insemination [36] and influence patterns of cytokine release in human cervical epithelial cells [41]. Together, the arrays of active seminal constituents, spermatozoa and semen extender initiate several immune mechanisms with immediate, mid-term and long-term effects on ovulation, sperm selection, fertilization and pregnancy outcome [13], which in its complexity is yet only partially understood.

## Conclusion

In conclusion, our original hypothesis that SP modifies the immune-cytokine network in porcine pre-ovulatory follicles was essentially confirmed. Use of an animal model with single horn SP-infusion and contralateral PBS-infusion as control revealed that SP orchestrates the gene network regulating the bidirectional communication between oocytes and surrounding somatic cells. SP initiated a particular response in the oocyte gene network of maturation associated transcripts pointing to a biological pathway by which SP may accelerate ovulation. This specific effect of porcine male genital fluid provides an effective mechanism to diminish sperm senescence in vivo. The female response is time dependent and influenced by the superordinate hormonal status. The primary key event seems to be the downregulation of *PTGS2* mRNA expression in the uterus, indicating a basic immunosuppressive mechanism which might counteract the inflammatory reaction caused by foreign cells and a volume effect of the inseminate. This study presents novel perspectives for the role of seminal fluid in modulation of female reproductive physiology. Knowledge in this area is important for developing future technologies in assisted reproduction in animals and human.

## Supporting information

S1 Data set (XLSX)

S1 Table Primer pairs uterus, granulosa, cumulus cells. (DOCX)

S2 Table Primer pairs oocytes. (DOCX)

S3 Table Summary mRNA expression animals. (DOCX)

S4 Table Correlation mRNA expression granulosa and cumulus cells. (DOCX)

S5 Table mRNA expression oocytes (means). (DOCX)
