# Supplementary material for: Seminal plasma modulates the immune-cytokine network in the porcine uterine tissue and pre-ovulatory follicles
Source: PLoS One. 2018 Aug 28;13(8):e0202654. doi: 10.1371/journal.pone.0202654 (PMC6112639; doi:10.1371/journal.pone.0202654)
Supplement: S1 Table — (DOCX) [file pone.0202654.s002.docx]

S1 Table:

Primer pairs used for RT-qPCR of uterine epithelium, granulosa cells and cumulus cells

| Gene | forward- (for) and reverse- (rev) primer(5’ 🡪 3’) | | Length (bp) | Reference |
| --- | --- | --- | --- | --- |
| PTGS2 | for | ATG ATC TAC CCG CCT CAC AC | 285 | Blitek et al. 2006 mod. |
|  | rev | GAA AAG CAG CTC TGG GTC AAA C |  |  |
| IL6 | for | TGG ATA AGC TGC AGT CAC AGA AC | 108 | Moue et al. 2008 mod. |
|  | rev | ATC CGA ATG GCC CTC AGG C |  |  |
| PPARG1 | for | GCC AAC TCT AAG CCA CTA ACA TAC AG | 73 | Lord et al. 2006 |
|  | rev | TGG TCA CCT CGC TAA AAG ATG A |  |  |
| PTX3 | for | TCC CCT GGA ACG TGG ACC CAT | 115 | Acc. No^a^ GQ_412351 |
|  | rev | TGT GAC CCG TGG CCA TGT CG |  |  |
| TNFA | for | CTC TTC TGC CTA CTG CAC TTC | 149 | Taylor et al. 2009 mod. |
|  | rev | GCT TTG ACA TTG GCT ACA ACG TG |  |  |
| TNFAIP6 | for | AAC AAG CGG CAG GCG TGT ACC | 104 | Acc. No^a^ NM_001159607 |
|  | rev | GGC AAG ACG GCC ACC TTC GT |  |  |
| UBB | for | GTC TGA GGG GTG GCT GCT AA | 85 | Acc. No^a^ NM_001105309 |
|  | rev | TGG GGC AAA TGG CTA GAG TG |  |  |

^a^ Acc. No: Accession number of the nucleotide-sequence of the NCBI gene-databank, which was used for the generation of the primers
