# Supplementary material for: Seminal plasma modulates the immune-cytokine network in the porcine uterine tissue and pre-ovulatory follicles
Source: PLoS One. 2018 Aug 28;13(8):e0202654. doi: 10.1371/journal.pone.0202654 (PMC6112639; doi:10.1371/journal.pone.0202654)
Supplement: S2 Table — (DOCX) [file pone.0202654.s003.docx]

S2 Table:

Primer pairs used for RT-qPCR of oocytes

| Gene | forward- (for) and reverse- (rev) primer (5’ 🡪 3’) | | length (bp) | Reference |
| --- | --- | --- | --- | --- |
| BMP15 | for | ATT TCA TCC CTG GAC ACT GC | 203 | Acc. No^a^ NM_001005155 |
| BMP15 | rev | TGG TTA CTT TCA GGC CCA TC |  |  |
| CDK1 | for | CCT GCA AGG GAT TGT GTT TT | 201 | Acc. No^a^ NM_001159304 |
| CDK1 | rev | GCT GAC CCC AGC AAT ACT TC |  |  |
| Cyclin B1 | for | TTG GTG TCA CTG CCA TGT TT | 200 | Acc. No^a^ NM_001170768 |
| Cyclin B1 | rev | TCC AAT CTT GGA TGC TCT CC |  |  |
| c-Mos | for | AAA TCA GCG ACT TTG GTT G | 200 | Acc. No^a^ NM_001113219 |
| c-Mos | rev | CTG ACG CTC CCC TGA GTA AG |  |  |
| GDF9 | for | CCC CTA GTG GTC TCC AAC AA | 193 | Acc. No^a^ NM_001001909 |
| GDF9 | rev | TTT TCC AGG GGA GGG AAT AC |  |  |
| MAPK1 | for | GAT GAC TTG CCC AAG GAA AA | 201 | Acc. No^a^ XM_001929509 |
| MAPK1 | rev | GGC TCA AAG GAG TCA AGG TG |  |  |
| PCNA | for | TGT GCT GGC AAT GAA GAC AT | 209 | Acc. No^a^ DQ473295 |
| PCNA | rev | TCT CGG CAT ATA CGT GCA AA |  |  |
| ZAR1 | for | CCT ACG TGT GGT GTG TCC AG | 201 | Acc. No^a^ NM_001129956 |
| ZAR1 | rev | CTT TGC ATC TCC CAC ACA AA |  |  |
| Globin | for | GCA GCC ACG GTG GCG AGT AT | 257 | Acc. No^a^ X04751 |
| Globin | rev | GTG GGA CAG GAG CTT GAA AT |  |  |

^a^ Acc. No: Accession number of the nucleotide-sequence of the NCBI gene-databank, which was used for the generation of the primers
