# Supplementary material for: Seminal plasma modulates the immune-cytokine network in the porcine uterine tissue and pre-ovulatory follicles
Source: PLoS One. 2018 Aug 28;13(8):e0202654. doi: 10.1371/journal.pone.0202654 (PMC6112639; doi:10.1371/journal.pone.0202654)
Supplement: S3 Table — (DOCX) [file pone.0202654.s004.docx]

S3 Table:

Messenger RNA expression of selected transcripts at different locations of the reproductive tract after single uterine horn infusion of seminal plasma and contralateral infusion of PBS.

| Localisation | Uterus | UTJ | Granulosa cells | Cumulus cells |
| --- | --- | --- | --- | --- |
| Gilts (n) | 16 | 16 | 16 | 14 |
| Transcript | SP/C (n) | SP/C (n) | SP/C (n) | SP/C (n) |
| PTGS2 | 16/16 | 14/14 | 14/15 | 14/13 |
| IL6 | 11/12 | 6/10 | 6/6 | 0/0 |
| PPARG1 | 0/0 | 0/0 | 16/16 | 5/5 |
| PTX3 | 2/4 | 5/5 | 15/14 | 14/14 |
| TNFA | 15/16 | 12/12 | 0/0 | 0/0 |
| TNFAIP6 | 15/15 | 15/15 | 12/12 | 11/11 |
| UBB | 16/16 | 16/16 | 16/16 | 14/14 |

|  | mRNA-expression > 100 copies in the majority of animals | |
| --- | --- | --- |
|  |  | |
| SP/C | | Number of gilts with mRNA expression ipsilateral of uterine infusion of  seminal plasma (SP) and contralateral (PBS control, C) |
