# Supplementary material for: Seminal plasma modulates the immune-cytokine network in the porcine uterine tissue and pre-ovulatory follicles
Source: PLoS One. 2018 Aug 28;13(8):e0202654. doi: 10.1371/journal.pone.0202654 (PMC6112639; doi:10.1371/journal.pone.0202654)
Supplement: S4 Table — (DOCX) [file pone.0202654.s005.docx]

S4 Table

Correlations (r, p<0.05) between mRNA expression levels of transcripts in granulosa and cumulus cells after
a single uterine horn infusion of seminal plasma on ipsilateral (SP) and contralateral sides (C)

1. Group 1: 2 h after treatment (n = 9 gilts)

|  |  | Granulosa cells | | | | | | Cumulus cells | | | | | |
| --- | --- | --- | --- | --- | --- | --- | --- | --- | --- | --- | --- | --- | --- |
|  |  | PTGS2 | | | PTX3 | | | PTGS2 | | | PTX3 | | |
| Granulosa cells | | SP | C | SP | | C | SP | | C | SP | | C |  |
| PTGS2 | SP |  |  | n.s. | |  | n.s. | |  | n.s. | |  |  |
|  | C |  |  |  | | n.s. |  | | n.s. |  | | n.s. |  |
| PTX3 | SP | 0.99 |  |  | |  | 0.821 | |  | n.s. | |  |  |
|  | C | n.s. | 0.90 |  | |  |  | | n.s. |  | | n.s. |  |
| Cumulus cells | |  |  |  | |  |  | |  |  | |  |  |
| PTGS2 | SP | n.s. |  | n.s. | |  |  | |  |  | |  |  |
|  | C |  | n.s. |  | | n.s. |  | |  |  | |  |  |
| PTX3 | SP | n.s. |  | n.s. | |  | 0.929 | |  |  | |  |  |
|  | C |  | n.s. |  | | n.s. |  | | 0.821 |  | |  |  |

1. Group 2 Group 1: 17 h after treatment (n = 7 gilts)

|  |  | Granulosa cells | | | | | | Cumulus cells | | | | | |
| --- | --- | --- | --- | --- | --- | --- | --- | --- | --- | --- | --- | --- | --- |
|  |  | PTGS2 | | | PTX3 | | | PTGS2 | | | PTX3 | | |
| Granulosa cells | | SP | C | SP | | C | SP | | C | SP | | C |  |
| PTGS2 | SP |  |  | n.s. | |  | n.s. | |  | n.s. | |  |  |
|  | C |  |  |  | | n.s. |  | | n.s. |  | | n.s. |  |
| PTX3 | SP | 0.86 |  |  | |  | 0.86 | |  | n.s. | |  |  |
|  | C | n.s. | 0.82 |  | |  |  | | 0.82 |  | | n.s. |  |
| Cumulus cells | |  |  |  | |  |  | |  |  | |  |  |
| PTGS2 | SP | n.s. |  | n.s. | |  |  | |  |  | |  |  |
|  | C |  | n.s. |  | | n.s. |  | |  |  | |  |  |
| PTX3 | SP | n.s. |  | n.s. | |  | n.s. | |  |  | |  |  |
|  | C |  | n.s. |  | | n.s. |  | | n.s. |  | |  |  |
