# Supplementary material for: Seminal plasma modulates the immune-cytokine network in the porcine uterine tissue and pre-ovulatory follicles
Source: PLoS One. 2018 Aug 28;13(8):e0202654. doi: 10.1371/journal.pone.0202654 (PMC6112639; doi:10.1371/journal.pone.0202654)
Supplement: S5 Table — (DOCX) [file pone.0202654.s006.docx]

S5 Table:

Relative amounts of transcript mRNA expression in oocytes retrieved from gilts at 2 h (A) and 17 h (B) after single uterine horn infusion of seminal plasma and contralateral infusion of PBS. SP: Oocytes collected from ipsilateral ovaries. C: oocytes collected from contralateral ovaries. Data are expressed as means ± SEM of relative to the mRNA content of external standard globulin. Values between SP and C did not differ significantly (p>0.05).

A) Group 1: 2 h after treatment, n = 9 gilts

| ZAR 1 | | c-Mos | | MAPK1 | | Cyclin B1 | | CDK1 | | PCNA | | GDF9 | | BMP15 | |
| --- | --- | --- | --- | --- | --- | --- | --- | --- | --- | --- | --- | --- | --- | --- | --- |
| SP | C | SP | C | SP | C | SP | C | SP | C | SP | C | SP | C | SP | C |
| 341 | 318 | 95 | 38 | 14 | 14 | 88 | 58 | 32 | 20 | 340 | 216 | 71 | 55 | 377 | 296 |
| ± 121 | ± 97 | ± 44 | ± 14 | ± 8 | ± 8 | ± 38 | ± 23 | ± 12 | ± 7 | ± 119 | ± 71 | ± 24 | ± 17 | ± 119 | ± 94 |

B) Group 2: 17 h after treatment, n = 7 gilts

| ZAR 1 | | c-Mos | | MAPK1 | | Cyclin B1 | | CDK1 | | PCNA | | GDF9 | | BMP15 | |
| --- | --- | --- | --- | --- | --- | --- | --- | --- | --- | --- | --- | --- | --- | --- | --- |
| SP | C | SP | C | SP | C | SP | C | SP | C | SP | C | SP | C | SP | C |
| 529 | 265 | 83 | 60 | 19 | 32 | 87 | 79 | 34 | 27 | 271 | 337 | 82 | 52 | 570 | 228 |
| ± 326 | ± 130 | ± 44 | ± 24 | ± 12 | ± 28 | ± 36 | ± 32 | ± 14 | ± 8 | ± 90 | ± 98 | ± 41 | ± 16 | ± 344 | ± 62 |
